# Short-hairpin RNA library: identification of therapeutic partners for gefitinib-resistant non-small cell lung cancer

**DOI:** 10.18632/oncotarget.2891

**Published:** 2014-11-25

**Authors:** Makoto Sudo, Seiichi Mori, Vikas Madan, Henry Yang, Geraldine Leong, H. Phillip Koeffler

**Affiliations:** ^1^ Cancer Science Institute of Singapore, NUS, Singapore; ^2^ Division of Cancer Genomics, The Cancer Institute of Japanese Foundation for Cancer Research, Tokyo, Japan; ^3^ Department of Hematology and Oncology, Cedars-Sinai Medical Center, Los Angeles, CA, USA; ^4^ National University Cancer Institute, National University Hospital, Singapore

**Keywords:** combination chemotherapy, dasatinib, gefitinib, PRKCSH, short-hairpin RNA library screening, thioridazine

## Abstract

Somatic mutations of the epidermal growth factor receptor often cause resistance to therapy with tyrosine kinase inhibitor in non-small cell lung cancer (NSCLC). In this study, we aimed to identify partner drugs and pathways that can induce cell death in combination with gefitinib in NSCLC cells. We undertook a genome-wide RNAi screen to identify synthetic lethality with gefitinib in tyrosine kinase inhibitor resistant cells. The screening data were utilized in different approaches. Firstly, we identified *PRKCSH* as a candidate gene, silencing of which induces apoptosis of NSCLC cells treated with gefitinib. Next, in an *in silico* gene signature pathway analysis of shRNA library data, a strong correlation of genes involved in the CD27 signaling cascade was observed. We showed that the combination of dasatinib (NF-κB pathway inhibitor) with gefitinib synergistically inhibited the growth of NSCLC cells. Lastly, utilizing the Connectivity Map, thioridazine was identified as a top pharmaceutical perturbagen. In our experiments, it synergized with gefitinib to reduce p-Akt levels and to induce apoptosis in NSCLC cells. Taken together, a pooled short-hairpin library screen identified several potential pathways and drugs that can be therapeutic targets for gefitinib resistant NSCLC.

## INTRODUCTION

Initial broad-based clinical studies found that only a minority of individuals with non-small cell lung cancer (NSCLC) responded to either gefitinib or erlotinib, which are tyrosine kinase inhibitors (TKIs) blocking activation of the epidermal growth factor (EGFR) [1-4]. Asian, non-smoking females were noted to be particularly sensitive to this class of drugs [2, 5]. Subsequently, investigators discovered that sensitivity to these TKIs was correlated with the presence of somatic mutations that affect the kinase domain of EGFR, especially either deletions within exon 19 or a L858R mutation in exon 21 [6-8]. Individuals with these tumors often respond to TKI therapy, but usually have progressive disease after 6-12 months of therapy. These resistant tumors frequently acquired either an additional mutation (T790M) in exon 20 of EGFR or a second mutation in the downstream pathway of EGFR, both resulting in lack of response to EGFR-TKI [9-11]. Other mechanisms of resistance include either amplification of *c-met* [12, 13] or deletion of *PTEN* [14, 15]. A major therapeutic priority is to identify drugs that can reverse the TKI resistance in exon 20 mutant EGFR. In order to discover additional drugs and pathways that might inhibit growth of NSCLC with an exon 20 mutant EGFR, we screened with a short-hairpin library for synergism with gefitinib [16-20] and identified several molecular biological pathways and pharmaceutical compounds that inhibited these TKI-resistant NSCLC cells.

## RESULTS

**Fig.1:**
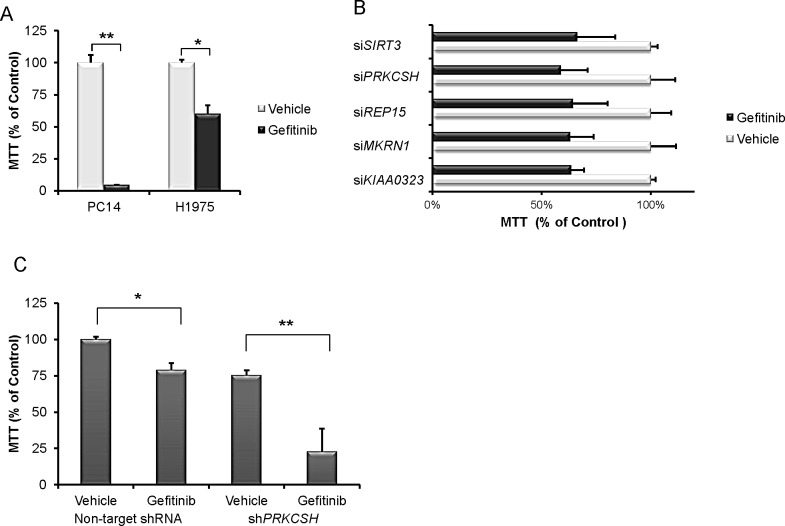

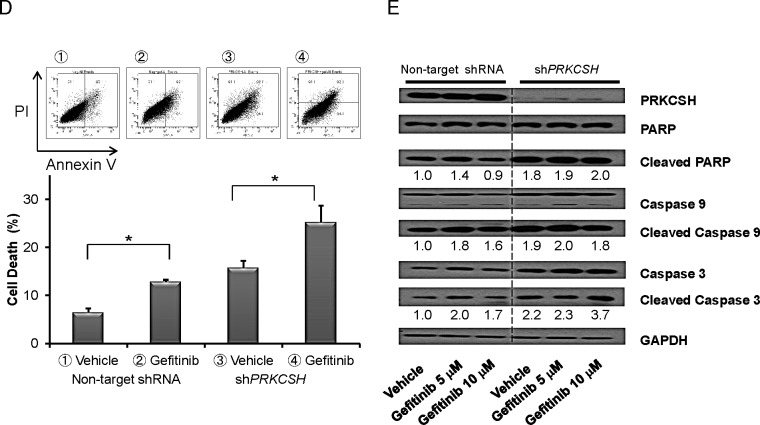
PRKCSH knockdown cells have increased sensitivity to gefitinib (A) MTT assay was performed using H1975 NSCLC cells [TKI-resistant, EGFR exon 20 (T790M) and exon 21 mutation] and PC14 NSCLC cells [TKI-sensitive, EGFR exon 19 (delE746-A750)]. Cells were treated with either vehicle or 10 μM gefitinib for 3 days. Data represent the mean and SD of experiments done in triplicate. (B) H1975 cells were cultured with either vehicle or 10 μM gefitinib after transduction with either a non-target siRNA or gene-specific siRNA. Relative cell viability (vehicle – gefitinib) / non-target siRNA vehicle x 100 % was measured. (C) H1975 NSCLC with stable silencing of PRKCSH (see panel E) were cultured for 3 days either with or without gefitinib (10 μM) and cell proliferation was measured (MTT assay). (D) H1975 NSCLC cells either with or without stable silencing of PRKCSH (see panel E) were cultured for 6 days either with or without gefitinib (10 μM) and total cell death [apoptosis and necrosis (Annexin V^+^)] (%) was measured. (E) H1975 NSCLC cells transduced with either a non-target shRNA or a stable PRKCSH shRNA were cultured with either 5 or 10 μM gefitinib for 24 hours. Lysates were western blotted and probed with antibodies against either PRKCSH, PARP, cleaved PARP, caspase 9, cleaved caspase 9, caspase 3, cleaved caspase 3 and GAPDH (loading control). Densitometry of bands of cleaved PARP, cleaved caspase 9, cleaved caspase 3 and GAPDH was performed with image J software. Band intensities were normalized to GAPDH band intensity. * *p* < 0.05, ** *p* value < 0.01.

The focus of our study was to identify target genes and/or agents that can overcome TKI-resistance in NSCLC. Initial growth curves (MTT assays) showed that H1975 NSCLC cells [TKI-resistant, harbors EGFR exon 20 (T790M) and exon 21 (L858R) mutations] was more resistant to gefitinib than PC14 cells [TKI-sensitive, EGFR exon 19 (delE746-A750)] (Fig. 1A). A screen was conducted for genes that, when silenced, enhanced TKI sensitivity in H1975 cells. The cells were transduced with a small hairpin RNAs (shRNAs) pooled library and a synthetic lethality screen in the presence of gefitinib was performed. Hairpins targeting 35 genes (from 16,000 genes) reproducibly conferred gefitinib sensitivity in H1975 cells (threshold relative fold depletion in gefitinib versus vehicle as log2 ratio and *p*-value < 0.05; Table 1).

**Table 1:** List of shRNA primary screen hairpin hits in gefitinib-treated NSCLC cells

Screening Hit	log2 (gefitinib/vehicle)	*p*-value
HIST1H4B	-7.25624	0.00010
ATAD3A	-6.6673613	0.00016
MKRN1	-7.2719342	0.00271
SLC12A6	-3.272012	0.00434
C9orf106	-4.0508964	0.00614
PDE6G	-5.233921	0.00615
TBX22	-3.3274011	0.00931
KIAA0323	-2.5301581	0.00972
PMS2L2	-6.6600143	0.01012
IFNA6	-6.4874721	0.01209
C16orf53	-2.5146758	0.01228
PARVA	-6.776589	0.01379
EPHX2	-4.0295129	0.01418
EPHX3	-4.8226394	0.01602
PRKCSH	-3.0505045	0.01675
SNX2	-5.6360098	0.01756
SERINC3	-3.2701922	0.01983
SLC25A14	-5.9946208	0.02349
C2CD2	-1.8099412	0.02931
MS4A4A	-10.767529	0.03117
LONP2	-4.9167331	0.03160
COG8	-5.7339535	0.03191
GPR56	-3.1508532	0.03728
APOBEC3F	-3.4803015	0.03769
HIAT1	-2.512018	0.03920
ATP5D	-5.6832087	0.04082
ZNF37A	-5.0617658	0.04344
SCARA5	-3.415674	0.03316
USP17L1P	-2.563753	0.03354
TUBGCP4	-2.8596609	0.03380
ALPP	-2.1846563	0.03579
SIRT3	-3.1603511	0.03581
EIF3C	-2.4709052	0.03622
MERTK	-1.8012646	0.04659
C2orf55	-2.2108206	0.04733

H1975 cells treated with either gefitinib or vehicle were infected with a pooled shRNA library consisting of 80,000 hairpins.

Primary screen hairpin hits displayed as relative fold depletion in gefitinib versus vehicle expressed as log2 ratio and *p*-value <0.05.

Twenty genes from the initial 35 target genes identified in the primary screen were examined for their growth inhibitory effects in gefitinib treated H1975 cells using independent siRNAs. Five genes (*SIRT3*, *PRKCSH*, *REP15*, *MKRN1*, *KIAA0323*) were confirmed to enhance the gefitinib-induced inhibition of cell proliferation (Fig. 1B). Silencing the *PRKCSH* gene produced the most profound growth inhibition. Therefore, expression of the *PRKCSH* gene was stably silenced in H1975 cells using lentiviral shRNA vector which markedly enhanced the gefitinib-related growth inhibition (50% decrease compared to gefitinib alone) and apoptosis (27% versus 17% with gefitinib alone) (Figs. 1C, D). Silencing of *PRKCSH* enhanced the expression of cleaved PARP, caspase-9 and -3 which was only slightly enhanced by addition of gefitinib (5, 10 μM) (Fig. 1E).

**Fig.2:**
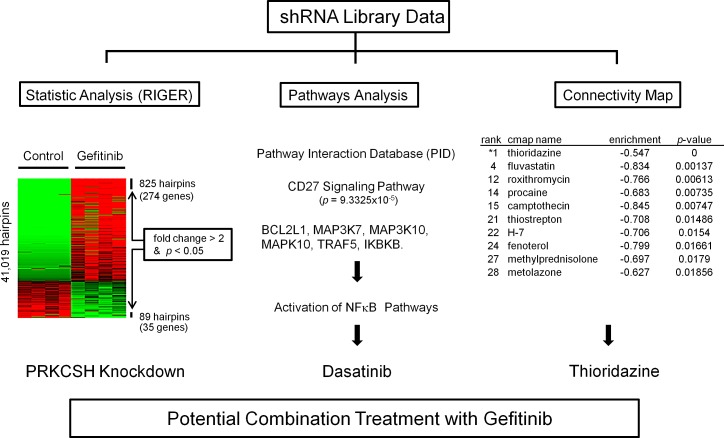
Three different approaches to identify a pathway or drug that can overcome gefitinib resistance of NSCLC H1975 cells were transduced with shRNA pooled library (MISSION^®^ shRNA) and a synthetic lethality screen with gefitinib was performed. shRNA library data were statistically analyzed by RIGER software (left panel). “Heat-map”: red and green represent either accumulation or depletion of the shRNA, respectively, when comparing the gefitinib treated and untreated cohort of H1975 NSCLC cells. Arrows point to those shRNA vectors whose abundance was significantly changed compared to control. Pathway analysis of the shRNA screen was done using NCI / Nature Pathway Interaction Database (PID) (middle panel). Connectivity Map software (right panel) was used to identify small-molecule combination therapy with gefitinib based on shRNA library data. All three approaches found effective combination treatment with gefitinib.

We next investigated which pathways were linked to gefitinib-induced cell death based on shRNA library data using NCI / Nature Pathway Interaction Database (PID) (http://pid.nci.nih.gav) (Table 2) [21]. The CD27 signaling pathway (including *BCL2L1, MAP3K7, MAP3K10, MAPK10, TRAF5, IKBKB*) was identified as the most significantly affected (*p*-value: 9.3325 E-05; Fig. 2, Table 2). This pathway relates to activation of NF-κB and pro-survival signaling. To explore the role of NF-κB pathway in gefitinib-induced cell death, cells were treated with dasatinib. Dasatinib is a known inhibitor of multiple tyrosine kinases (BCR-ABL, SRC family, c-Kit etc) which inhibits the NF-κB pathway upstream of Src pathway [22, 23].

**Table 2:** Analysis of gefitinib-treatment related signaling pathway in NSCLC cells

Pathway	Genes involved	*p-* value
CD27 Signaling	BCL2L1, MAP3K7, MAP3K10, MAPK10, TRAF5, IKBKB	9.33E-05
Reelin Signaling	MAPK10, MAP3K10, MAPK8IP2, MAPT, PAFAH1B3	0.005888
RANK Signaling	MAP3K7, MAP3K10, MAPK10, TRAF5, IKBKB	0.007413
Eicosanoid Signaling	ALOX12B, PLA2G4A, PTGER3, PTGIS	0.008299
Induction of Apoptosis	BCL2L1, MAPK10, IKBKB, SLC25A5	0.01
IL6 Signaling	CSNK2A1, MAP3K7, IL1RL2, MAPK10, IKBKB	0.009333
CD40 Signaling	MAP3K7, MAPK10, TRAF5, IKBKB	0.010965
April Mediated Signaling	MAPK10, TRAF5, IKBKB	0.011749
DNA Double-Stranded Break Repair by Homologous Recombination	GEN1, MRE11A	0.014791
p38 MAPK Signaling	DUSP1, HMGN1, IL1RL2, MAP3K7, PLA2G4A	0.016218
B Cell Activating Factors Signaling	MAPK10, TRAF5, IKBKB	0.018197
NF-kB Signaling	CSNK2A1, IKBKB, MAP3K7, TLR5, TRAF5	0.023442
Toll-like Receptor Signaling	IKBKB, MAP3K7, TLR5	0.026303
FXR/RXR Activation	FOXA3, HNF4A, MAPK10	0.02884

Gefitinib-treatment related signaling pathway was analyzed to identify new candidate inhibitors. shRNA primary hairpin screen” hits” were analyzed by using NCI / Nature Pathway Interaction Database (PID).

**Fig.3:**
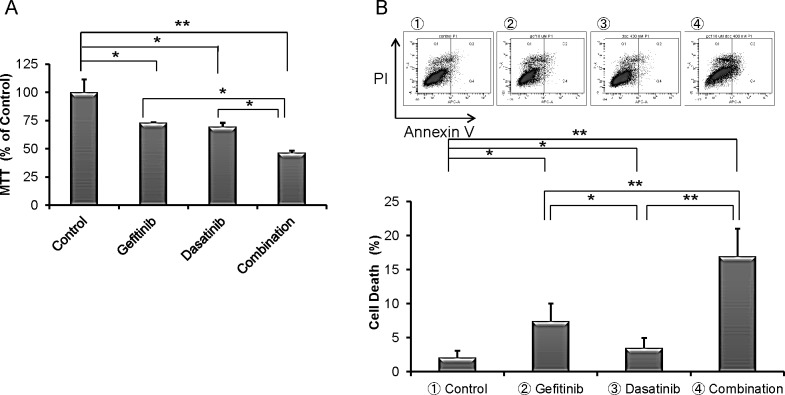

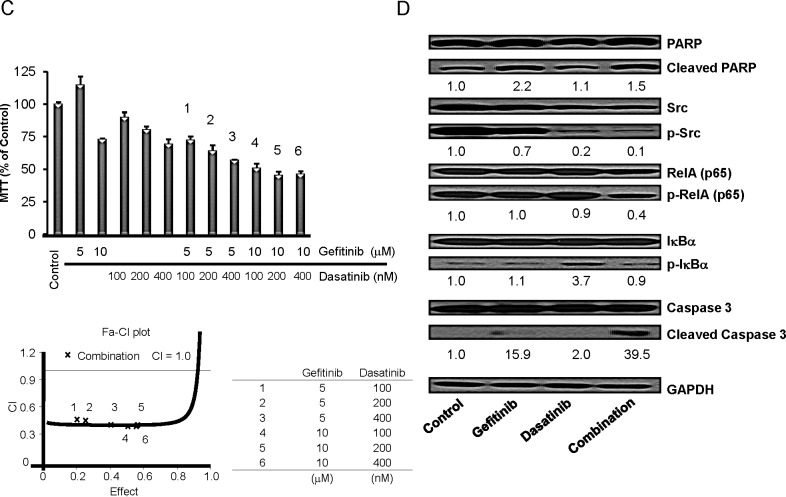
Combination of dasatinib and gefitinib synergistically inhibited cell proliferation of TKI-resistant H1975 NSCLC cells (A) Co-treatment with gefitinib (10 μM) and dasatinib (400 nM) for 3 days inhibited growth of H1975 cells greater than either inhibitor alone (MTT assays, mean ± SD of 3 experiments done in quadruplicate). (B) H1975 cells were cultured with gefitinib (10 μM), dasatinib (400 nM) and combination (gefitinib 10 μM, dasatinib 400 nM) for 6 days; cells were harvested and stained with Annexin-V and PI, and analyzed by flow cytometry. Cells in lower right quadrant (Annexin-V^+^/PI^−^) was defined as apoptotic cells and upper right quadrant (Annexin-V^+^/PI^+^) was defined as necrotic cells. Total cell death (%) [apoptotic cells and necrotic cells] was measured. Representative results are shown. (C) H1975 cells were exposed to gefitinib alone (5 μM or 10 μM), dasatinib alone (100, 200 or 400 nM) and combination treatment gefitinib (5 μM or 10 μM) and dasatinib (100, 200 or 400 nM) for 3 days. Combination treatment inhibited growth of H1975 cells greater than either inhibitor alone. Combination effect was measured by Calcuysin software. CI < 1, CI = 1 and CI > 1 represent synergism, additive, and antagonism of the two compounds, respectively. (D) H1975 cells were exposed to gefitinib (10 μM), dasatinib (400 nM) and combination (gefitinib 10 μM and dasatinib 400 nM) for 24 hours. Lysates were western blotted and probed with antibody against PARP, cleaved PARP, Src, phospho-Src, caspase 3, cleaved caspase 3 and GAPDH (loading control). Densitometry of bands of cleaved PARP, phospho-Src, cleaved caspase 3 and GAPDH was done with image J software, and band intensities were normalized to GAPDH band intensity. * *p* < 0.05, ** *p* value < 0.01.

Treatment of cells with dasatinib promoted gefitinib-induced cell inhibition of proliferation of NSCLC (Fig. 3A). Dasatinib treatment alone did not have a potent ability to induce cell death of H1975 cells; but when combined with gefitinib, they significantly increased cell death (Fig. 3B). Our studies using multiple concentration of gefitinib and dasatinib showed that co-treatment with gefitinib (5 or 10 μM) and dasatinib (100, 200 or 400 nM) synergistically inhibited growth of H1975 cells (CI<1 1-6 columns, Fig. 3C). Also, in contrast to either drug alone, this drug combination induced apoptosis, as shown by an increase in caspase 3 cleavage (Fig. 3D). Exposure to dasatinib attenuated phospho-Src, as well as phospho-Rel A (Fig. 3D).

**Fig.4:**
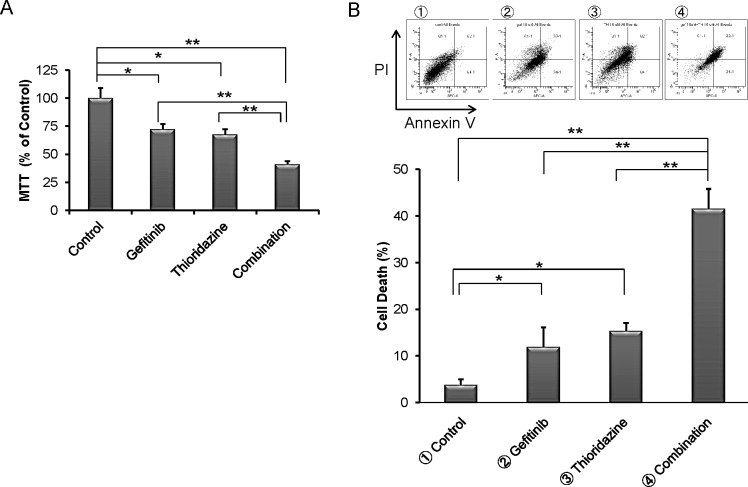

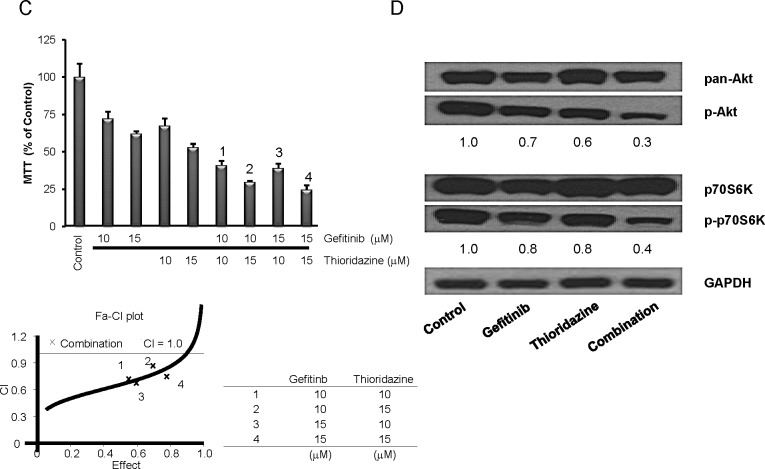
Effect of the combination of thioridazine and gefitinib on TKI-resistant NSCLC cells (A) Incubation with gefitinib (10 μM) and thioridazine (10 μM) for 3 days inhibited growth (MTT assay) of H1975 NSCLC cells greater than either inhibitor alone (MTT assays, mean ± SD of 3 experiments done in quadruplicate). (B) H1975 NSCLC cells were exposed to either gefitinib (10 μM) or thioridazine (10 μM), or their combination (gefitinib 10 μM, thioridazine 10 μM) for 6 days; cells were harvested and stained with Annexin-V and PI, and analyzed by flow cytometry. Lower right quadrant (Annexin-V^+^/PI^−^) was defined as apoptotic cells and upper right quadrant (Annexin-V^+^/PI^+^) was defined as necrotic cells. Total cell death (%) [apoptotic cells and necrotic cells] is displayed graphically. Representative results are shown. (C) Incubation with gefitinib (10 μM or 15 μM) and/or thioridazine (10 μM or 15 μM) for 3 days inhibited growth of H1975 cells measured in MTT assay (top of panel C). Combination effect was measured by Calcuysin software. CI < 1, CI = 1 and CI > 1 represent synergism, additive, and antagonism of the two compounds, respectively. (D) H1975 cells were cultured with either gefitinib (10 μM) and/or thioridazine (10 μM) for 24 hours. Lysates were western blotted and probed with antibody against, pan-Akt, phospho-Akt, p70S6K, phospho-p70S6K and GAPDH (loading control). Densitometry of bands of phospho-Akt,, phospho-p70S6K, and GAPDH was done with image J software. Band intensities were normalized to GAPDH band intensity. * *p* < 0.05, ** *p* value < 0.01.

*In-silico* screening using the Connectivity Map of our shRNA library data identified drugs that could enhance cell kill by gefitinib (Overview, Fig. 2). In brief, this correlation-based pattern-matching software utilizes the input gene signatures from our shRNA library analysis by RIGER. Based on the degree of difference between gefitinib and the pharmaceutical perturbagen, a connectivity score is assigned, and the negative enrichment score is used to identify a perturbagen inducing a synergistic effect with gefitinib (Table 3). The top pharmaceutical compounds were examined. Surprisingly, the first compound on the list was “thioridazine”, which has been reported to have antiproliferative activity against tumor cells [24]. Exposure of H1975 cells to either thioridazine (10 μM) or gefitinib (10 μM) treatment produced an approximate 25% growth inhibition; when the two drugs were combined, an appropriate 65% decreased growth occurred (Fig. 4A). Similarly, the combination of the two drugs caused cell death (annexin V/propidium iodine positivity) of about 40% of the lung cancer cells (H1975) compared to either gefitinib (10 μM) or thioridazine (15 μM) alone (~ 12% and 14%, respectively) (Fig. 4B). Furthermore, co-treatment with thioridazine and gefitinib for 3 days synergistically inhibited the growth of the lung cancer cells (CI<1, columns labeled 1-4; (Fig. 4C,). Thioridazine (10 μM) and gefitinib (10 μM) alone (24 hours) did not affect expression of either p-Akt or p-p70S6K in lung cancer cells; but together, they reduced levels of these activated signaling pathways (Fig 4D).

**Table 3:** Connectivity Map: Potential therapeutic agents enhancing gefitinib activity

rank	cmap name	mean	n	enrichment <−0.5	*p*-value	specificity
1	thioridazine	−0.437	20	−0.547	0	0.086
4	fluvastatin	−0.558	4	−0.834	0.00137	0
12	roxithromycin	−0.532	4	−0.766	0.00613	0.0333
14	procaine	−0.462	5	−0.683	0.00735	0.0558
15	camptothecin	−0.549	3	−0.845	0.00747	0.1818
21	thiostrepton	−0.443	4	−0.708	0.01486	0.0556
22	H-7	−0.507	4	−0.706	0.0154	0.3712
24	fenoterol	−0.567	3	−0.799	0.01661	0.0196
27	methylprednisolone	−0.492	4	−0.697	0.0179	0.0279
28	metolazone	−0.395	5	−0.627	0.01856	0.0056
30	hexamethonium bromide	−0.436	5	−0.62	0.02117	0.0323
31	ampicillin	−0.421	4	−0.684	0.02186	0.0126
32	econazole	−0.382	4	−0.682	0.02234	0.1111
33	bromocriptine	−0.31	5	−0.615	0.02331	0.0526
35	ifenprodil	−0.377	4	−0.673	0.0255	0.0805
38	tolfenamic acid	−0.339	4	−0.671	0.02626	0.0177
40	clotrimazole	−0.409	5	−0.602	0.0283	0.0909
41	vanoxerine	−0.342	4	−0.665	0.02851	0.0417
49	rottlerin	−0.534	3	−0.745	0.03371	0.1261
50	nabumetone	−0.421	4	−0.647	0.03702	0.038

The Connectivity Map is correlation-based pattern-matching software. Data from shRNA primary hairpin screen hits were analyzed by the Connectivity Map. This ranking (left column) reflects the ability of the pharmaceutical perturbagens to induce a synergistic effect with gefitinib.

## DISCUSSION

Almost all patients with a newly diagnosed NSCLC with either an exon 19 or 21 mutation of EGFR have an initial response to TKI therapy. However, invariably after 6-12 months, the tumor becomes resistant to therapy, associated with additional genomic changes especially a T790M EGFR mutation. Similar to the lessons learned with HIV therapy, treatment of these NSCLC with more than one drug may prevent emergence of a drug-resistant clone. Therefore, we performed a synthetic lethality screen with gefitinib using a pooled short-hairpin RNA library in NSCLC cells to help identify pathways druggable in TKI resistant NSCLC. Furthermore, the shRNA library data was used for gefitinib-related pathway analysis and *in-silico* gene signature based examination.

From the shRNA library screen and siRNA validation experiments in NSCLC cells, we identified and validated *PRKCSH* as a candidate gene that synergizes with gefitinib (Fig. 1). *PRKCSH* encodes the non-catalytic β-subunit of glucosidase II (GIIβ). Glucosidase II facilitates protein translocation and “quality control” pathways of the endoplasmic reticulum (ER). Mutation of this gene is associated with autosomal dominant polycystic liver disease (PCLD) [25-27]. Silencing of *PRKCSH* in NSCLC cells caused decreased cell proliferation and increase in the proportion of apoptotic cells, and when combined with gefitinib both these effects were significantly enhanced (Figs. 1C and D). Identification of *PRKCSH* led us to examine *PKD2* [28-30]. Both these molecules are involved in autosomal dominant polycystic kidney and liver diseases, and *PRKCSH* acts as a chaperone-like molecule to regulate *PKD2* expression. *PKD2* controls the endoplasmic reticulum (ER) regulated Ca^2+^ mediated apoptosis by decreasing the Ca^2+^ concentration in the ER [26]. Silencing of *PKD2* in NSCLC cells augmented apoptosis when combined with gefitinib (Supplement Fig. 1B, C). In addition, *PKD2* knockdown inhibited activation of the ERK pathway, which is associated with cell survival (Supplement Fig. 1C). To the best of our knowledge, neither *PRKCSH* nor *PKD2* has been reported as therapeutic targets for NSCLC. The use of either *PRKCSH* or *PKD2* inhibitors in combination with gefitinib to treat NSCLC requires further study.

Our shRNA library screen also identified the CD27 signaling pathway which relates to activation of NF-κB and pro-survival signals (Fig. 2 and Table 2). Dasatinib can modulate the NF-κB pathway; but alone, the drug had little effect on either apoptosis or proliferation of NSCLC. However, the combination of dasatinib and gefitinib synergistically induced cell death and inhibited cell growth (Fig. 3). Taken together, these experiments suggest that the combination of NF-κB inhibitors and gefitinib may be therapeutically effective for EGFR-mutant NSCLC.

The Connectivity Map is a genomic screening tool for linking genes associated with a selected phenotype with potential therapeutic agents [31, 32]. To discover additional therapeutic compounds that may synergistically interact with gefitinib, the shRNA library data were analyzed using the Connectivity Map software. Thioridazine, a phenothiazine derivative, was one of the prominent “hits”. Several reports have noted that the drug can have anticancer activity [33-35]. The mechanism by which this is mediated is unclear, although it may occur by inhibiting the PI3K/Akt pathway. The combined treatment of thioridazine and gefitinib synergistically inhibited proliferation of NSCLC cells (Fig. 4C). Neither thioridazine nor gefitinib suppressed levels of activated Akt and p70S6K in NSCLC, but their combination reduced phospho-Akt and phospho-p70S6K levels in these cells (Fig. 4D). Phenothiazines can cause severe extrapyramidal side-effects such as tardive dyskinesia. Thus, further modification of this molecule should be explored to incorporate the anti-NSCLC effects without neurological toxicity. Alternatively, phenothiazines activate PP2A; we (unpublished data) and others [36] have shown that drugs that activate PP2A have anti-cancer activity. Novel activators of this phosphatase may synergize with gefitinib to inhibit growth of EGFR mutant NSCLC.

In summary, shRNA library screen identified therapeutic partners of gefitinib for treatment of EGFR mutant NSCLC including EGFR T790M. Furthermore, the shRNA library data were utilized for gene signature based *in-silico* query which identified *PRKCSH* as an interesting partner with gefitinib. Finally, the shRNA results were used to interrogate the Connectivity Map. This pathway analysis identified thioridazine as a synergistic partner of gefitinib. In summary, this study using several experimental approaches has identified exciting drugs and pathways for further study in EGFR mutant NSCLC.

## MATERIALS AND METHODS

### Reagents and NSCLC cell lines

The following compounds were used in this study: gefitinib; dasatinib [LC Laboratories (MA, USA)]; thioridazine (10-[21-methyl-2-piperidyl) ethyl]-2-methylthio-phenothiazine) [Sigma Aldrich (Singapore)]. All reagents were dissolved in dimethyl sulfoxide (DMSO) and the final concentration of DMSO never exceeded 0.1% in culture. Antibodies: Akt [36], phospho-Akt (Ser473), caspase-3, 8, 9, cleaved caspase-3, 8, 9, ERK, phospho-ERK, IκBα, phospho-IκBα, p70S6K, phospho-p70S6K (Thr421 / Ser424), PKD2, PRKCSH, PARP, cleaved-PARP, RelA, phospho-RelA, Src, phospho-Src, GAPDH were purchased from Cell Signaling (MA, USA).

The human NSCLC cell lines H1975 and PC14 were purchased from the American Type Culture Collection (Manassas, VA). These cells were cultured in RPMI 1640 supplemented with 10% fetal calf serum, 100 U/ml of penicillin, and 100 μg/ml of streptomycin. The H1975 cell line has an EGFR T790M mutations in exon 20, associated with gefitinib and erlotinib resistance. The PC14 cell line has an EGFR delE746-A750 mutation in exon 19, associated with TKI sensitivity.

### Pooled short-hairpin RNA library screen

H1975 cells were transduced in quadruplicates with a pooled shRNA library (MISSION^®^ shRNA), consisting of 80,000 hairpins targeting 16,000 genes at an MOI of 0.3 hairpins/cell. After 48 hours, puromycin was added to the media (5 μg/ml) to select for transduced cells.

Puromycin was continuously in the media until cells were harvested. Following selection, each replicate was treated with either vehicle or gefitinib (8 μM) for 1 week. This concentration of gefitinb was identified as the IC50 to H1975 cells [37]. Cells were harvested and genomic DNA was isolated both prior to drug treatment and at day 10. shRNA sequences were amplified by PCR using specific primers. The purified PCR products were subjected to high-throughput sequencing using Illumina Genome Analyzer IIx. The quality of the data was checked, normalized and statistically analyzed by RNAi gene enrichment ranking (RIGER) software [38]. Significant hits were defined as those altered by 2 fold, p<0.05

### *PRKCSH* and *PKD2* knockdown cells

Stably knockdown of the expression of the *PRKCSH* and *PKD2* genes in H1975 cells used lentiviral shRNA vectors. *PRKCSH* shRNA plasmid was kindly provided by Prof. Gard Walz (Renal Division, University Hospital Freiburg). The *PRKCSH* shRNA sequence (5′-CCGGAAGTTCAGTGCCATGAAGTATCT CGAGATACTTCATGGCACTGAACTTTT TTTG-3′) was cloned into pLKO.1 vector. *PKD2* shRNA plasmid was constructed according to selected sequences within the gene and cloned into pLKO.1 vector. (5′-CCGGCACGACCAACAGATACTATCTCGAGATAGTATCTGTTGGTCGTGTTTTTG­-3′). The negative control shRNA [SHC002 (MISSION^®^ shRNA)] was purchased from Sigma. Cells were transduced and selected with puromycin (2 μg / ml, 10 days). Silencing of the genes were confirmed by western blotting.

### Cell proliferation assays

NSCLC cells were seeded at a density of 10,000 per well in 96-well plates. After 24 hours, these cells were cultured with multiple concentrations of drugs for 72 hours. Cell proliferation was determined using CellTiter 96^®^ Non-Radioactive Cell Proliferation Assay (Promega) according to the manufacturer's instructions; and the plates were read by a fluorescence spectrometer. Half-maximal inhibitory concentrations (“IC50-values”) were determined from the images under the growth inhibition curves using Prism 4.0 software. Calcusyn software (Biosoft) was used to analyze combination drug treatment data. CI<1, CI=1 and CI>1 represent synergism, additive, and antagonism of the two compounds, respectively (Fig. 3C, Fig. 4C)

### Cell death assays

Cells were harvested and stained with Annexin-V and PI (BD Biosciences), and analyzed by a FACSCalibur flow cytometer (BD Sciences). Lower right quadrant (Annexin-V^+^/PI^−^) and upper right quadrant (Annexin-V^+^/PI^+^) were defined as apoptotic cells and necrotic cells, respectively.

### Western blot analysis

Cells were harvested, washed with PBS, and lysed in ProteoJET Mammalian Cell Lysis Regent (Fermentas) supplemented with ProteoBlock Protease Inhibitor Cocktail (Fermentas). Protein concentrations were measured by Bradford assay (Bio-Rad, Richmond, CA). Equal amounts of protein were dissolved in SDS-polyacrylamide gel electrophoresis sample loading buffer and electrophoresed in a polyacrylamide gel (7.5% or 10%). After electrophoresis, the proteins were electrotransferred to a polyvinylidene difluoride membrane (Immobilon, Millipore, Bedford, MA). Immunoblotting was performed using antibodies mentioned above, and detected by ECL-Plus reagent (Amersham, Boston, MA).

### Statistical analysis

Data were collected using a minimum of three experiments and used to calculate the mean ± S.D. Statistical significance was calculated using either ANOVA or student-t test and was considered significant at p values < 0.05.

## SUPPLEMENTARY MATERIAL AND FIGURE


