# Supplementary material for: Short-hairpin RNA library: identification of therapeutic partners for gefitinib-resistant non-small cell lung cancer
Source: Oncotarget. 2014 Nov 25;6(2):814–24. doi: 10.18632/oncotarget.2891 (PMC4359257; doi:10.18632/oncotarget.2891)
Supplement: Supplementary file 1 [file oncotarget-06-814-s001.pdf]

Short-hairpin RNA library: identification of therapeutic partners for gefitinib-resistant non-small cell lung cancer

Supplementary Material

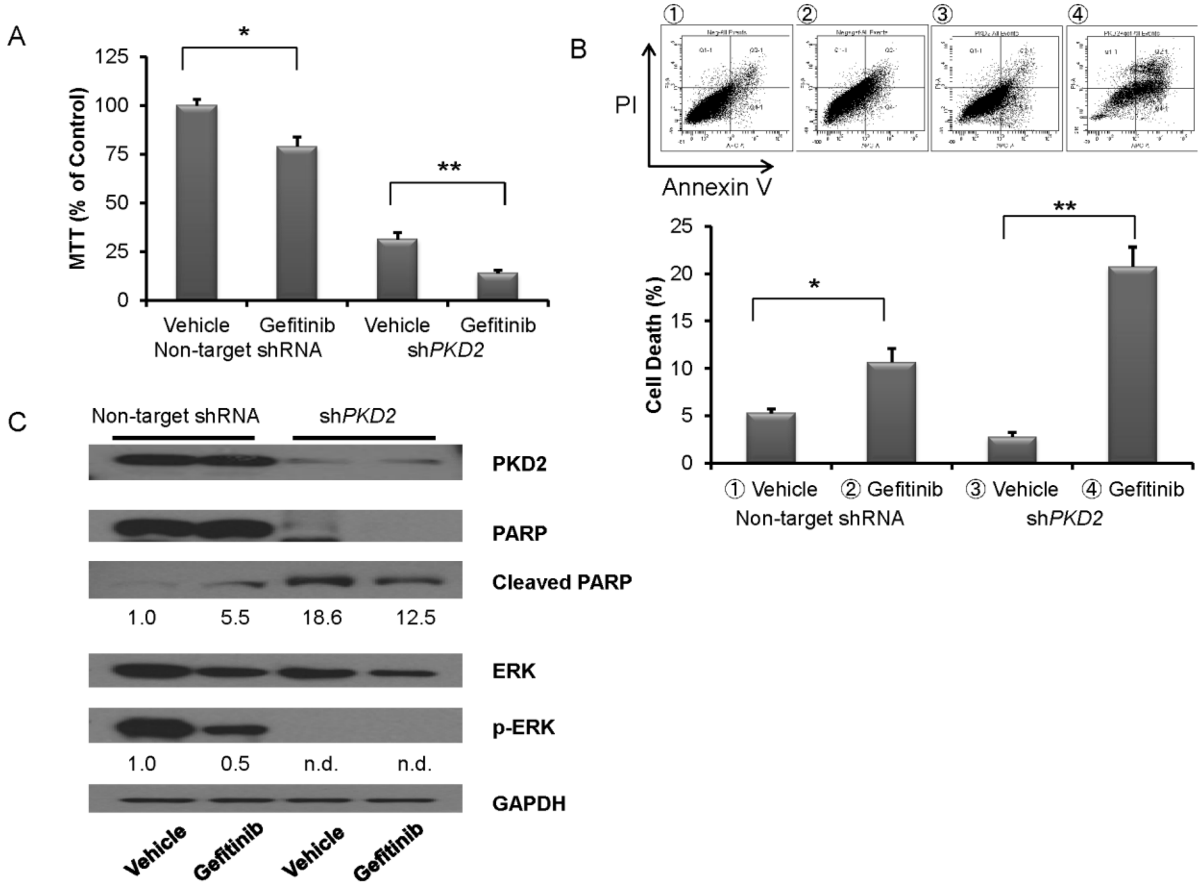

Supplement Fig.1: **Effect of silencing of PKD2 in H1975 NSCLC cells.** (A) H1975 NSCLC with stable silencing of PKD2 (see panel C) were cultured for 3 days either with or without gefitinib (10 µM) and cell proliferation was measured (MTT assay). (B) H1975 NSCLC cells either with or without stable silencing of PKD2 (see panel C) were cultured for 6 days either with or without gefitinib (10 µM); total cell death [apoptosis and necrosis (Annexin V^+)] (%) was measured. (C) H1975 NSCLC having either a scrambled shRNA or a stably integrated PKD2 shRNA were cultured with 10 µM gefitinib for 24 hours. Lysates were western blotted and probed with antibody against either PKD2, PARP, cleaved PARP, ERK, phospho-ERK and GAPDH (loading control). Densitometry of bands of cleaved PARP, phospho-ERK and GAPDH was done with image J software. Band intensities were normalized to GAPDH band intensity. * $p < 0.05$, ** p value < 0.01.
